# Associations between early childhood caries, malnutrition and anemia: a global perspective

**DOI:** 10.1186/s40795-020-00340-z

**Published:** 2020-05-04

**Authors:** Morenike Oluwatoyin Folayan, Maha El Tantawi, Robert J. Schroth, Ana Vukovic, Arthur Kemoli, Balgis Gaffar, Mary Obiyan, Early Childhood Caries Advocacy Group

**Affiliations:** 1 Department of Child Dental Health, Obafemi Awolowo University, Ile-Ife, Nigeria; 2 Department of Pediatric Dentistry and Dental Public Health, Faculty of Dentistry, Alexandria University, Alexandria, Egypt; 3 Department of Preventive Dental Science, Dr. Gerald Niznick College of Dentistry, University of Manitoba, Winnipeg, Canada; 4 Departments of Pediatrics and Child Health and Community Health Sciences, Max Rady College of Medicine, Rady Faculty of Health Sciences, University of Manitoba, Winnipeg, Canada; 5 Department of Pediatric and Preventive Dentistry, School of Dental Medicine, University of Belgrade, Belgrade, Serbia; 6 Department of Paediatric Dentistry and Orthodontics, University of Nairobi, Nairobi, Kenya; 7 Department of Preventive Dental Sciences, College of Dentistry, Imam Abdulrahman University, Dammam, Saudi Arabia; 8 Department of Demography and Social Statistics, Obafemi Awolowo University, Ile-Ife, Nigeria

**Keywords:** Stunting, Overweight, Underweight, Free sugar, Gross National Income per capita

## Abstract

**Background:**

Malnutrition is the main risk factor for most common communicable diseases. The aim of this study is to determine the relationship between country-level prevalence of early childhood caries (ECC), malnutrition and anemia in infants and preschool children.

**Methods:**

Matched country-level ECC, malnutrition and anemia prevalence were generated from databases covering the period 2000 to 2017. Multivariate general linear models were developed to assess the relationship between outcome variables (prevalence of stunting, wasting, overweight, and anemia) and the explanatory variable (ECC prevalence) adjusted for gross national income per capita. Adjusted regression coefficients (B) and partial eta squared were computed.

**Results:**

The mean (standard deviation (SD)) ECC prevalence was 23.8 (14.8)% for 0–2 year-olds and 57.3 (22.4)% for 3–5-year-olds. The mean (SD) prevalence of wasting was 6.3 (4.8)%, overweight 7.2 (4.9)%, stunting 24.3 (13.5)%, and anemia 37.8 (18.1)%. For 0–2-year-olds, the strongest and only significant association was between the prevalence of ECC and overweight (η2 = 0.21): 1 % higher ECC prevalence was associated with 0.12% higher prevalence of overweight (B = 0.12, *P* = 0.03). In 3–5-year-olds, the strongest and only significant association was between the prevalence of ECC and anemia (η2 = 0.08): 1 % higher prevalence of ECC was associated with 0.14% lower prevalence of anemia (B = − 0.14, *P* = 0.048).

**Conclusion:**

Country-level prevalence of ECC was associated with malnutrition in 0–2-year-olds and with anemia in 3–5-year-olds. The pathway for the direct relationship between ECC and overweight may be diet related. The pathway for the inverse relationship between ECC and anemia is less clear and needs further investigations.

## Background

There are global concerns about the multi-facet impact of malnutrition in children. The term malnutrition includes undernutrition (underweight, wasting, stunting, micronutrient deficiencies and low birth weight), which is present most often in disadvantaged communities, and over consumption of food (overweight and obesity), which is present in both developed and developing communities [1]. Malnutrition is the main risk factor for most common communicable diseases and contributes to acute deaths in children under five years of age [2]. On the other hand, overweight and obesity increase the risk for non-communicable diseases, poor adulthood health, and premature death [1].

Early childhood caries (ECC) is dental decay that affects the primary dentition in children younger than 6 years of age [3]. It is a challenging problem faced by infants, toddlers and preschool children in developed and developing countries [4]. Severe forms of ECC impact children’s growth, development, and well-being [5], and can have negative social and economic effects on parents and society [6]. Malnutrition has many of the same etiological factors as ECC, including poor dietary habits and food intake, and social-economic inequalities [7]. If not addressed, ECC can destroy the primary dentition causing oral pain that can interfere with eating and sleeping. These consequences can result in a child being underweight [8, 9] and stunted [10]. Also, vitamin D, iron, calcium and albumin deficiencies and protein-energy malnutrition may lead to enamel defects that make the enamel surface rough and prone to accumulation of plaque, with subsequent post-eruptive caries [11, 12]. Malnutrition can also cause salivary gland hypo-function, with reduced saliva flow rate and buffering capacity [13, 14], and change in salivary constituents ratio, particularly amylase, lysozyme, and immunoglobulins. These changes are associated with higher risk of caries [15–19]. Thus, the relationship between malnutrition and ECC is complex, bi-directional and involves co-morbid relationships [7].

Recent studies have highlighted the relationship between ECC and malnutrition [20], micronutrient deficiencies [11, 12, 21–23], and anemia (which may result from malnutrition [24]). However, most of the evidence on the relationship between ECC, malnutrition and anemia is inconclusive. To the best of our knowledge, the scientific literature lacks macro-level data on the relationship between these three public health issues that have shared etiological factors. Understanding these relationships might help in to designing cost-effective and efficient interventions using the common risk factor approach, and targeting at-risk children in parts of the world where the problems are most concentrated [20].

The purpose of this study was to determine the association between country-level prevalence of ECC with malnutrition and anemia in infants and preschool children. The null hypothesis of the study was that ECC prevalence in 0 to 2-year-olds and 3 to 5-year-olds is not associated with malnutrition or anemia.

## Methods

This was an ecologic study. We collected macro-level data about ECC, anemia and nutritional status of children under six years of age for the period January 2007 to October 2017 for United Nations member States [25].

### Data sources

#### Prevalence of ECC

According to the American Academy of Pediatric Dentistry, children < 72 months of age with one or more decayed, missing due to decay or filled primary tooth surfaces have ECC [3]. The data on ECC prevalence were extracted from the World Health Organization (WHO) Country Oral Health Profile database and other online databases. Most estimates of ECC were based on cavitated lesions only and they were included in the study data. No language filter was applied for the database search. The retrieved data were used to calculate the ECC prevalence for each country by dividing the total number of children affected with ECC by the total number of children examined multiplied by 100. Most retrieved studies did not provide estimates of ECC severity such as the number of affected teeth/surfaces or the number of teeth/surfaces that were filled or missing. Thus, the data used were only the prevalence of ECC prevalence. Prevalence was calculated for two separate age groups: 0–2 and 3–5-year-olds. More details on the computation of country level ECC prevalence were reported in our previous paper [26].

#### Nutritional status of children under-5 years

Information on nutritional status was obtained from country-level data jointly produced by the WHO, UNICEF and World Bank in 2018 [27, 28] covering the period 2000 to 2017. The thresholds for defining stunting, wasting and overweight were established through the WHO-UNICEF Technical Advisory Group on Nutrition Monitoring [27] and were developed in relation to standard deviations (SD) of the normative WHO Child Growth Standards. We used the following definitions applying to children aged 0–5 years old:

1. Stunting: below minus two SDs from median height-for-age.
2. Wasting: below minus two SDs from median weight-for-height.
3. Overweight: above one SD from median weight-for-height.

The prevalence of wasting, stunting and overweight were reported as the percentage of children 0–5 years old who met the definition.

#### Anemia status in children under-5 years of age

We used country-level estimates for anemia prevalence from the WHO [29], where iron-deficiency anemia was defined as blood hemoglobin concentrations < 110 g/l in children younger than 5 years of age.

### Data analysis

The data sets (ECC, malnutrition, and anemia indicators) were matched by country. Scatter plots were used to represent the correlation between the prevalence of anemia, malnutrition and ECC in the two age groups (0–2-year-olds and 3–5-year-olds), and correlation coefficients and *p* values were calculated. Multivariate analysis of variance (MANOVA) with the SPSS procedure multivariate general linear analysis was used to develop two separate models assessing the relationship between outcome variables (prevalence of types of malnutrition; and prevalence of anemia) and two explanatory variables (ECC prevalence) for each two-age group. Each model was adjusted for the economic level of the country according to the 2017 Gross National Income per capita calculated with the World Bank Atlas method [30] based on our previous finding of the association between global ECC prevalence and growth in per capita gross national income [26]. The groups were: low income ($995 or less); lower middle income ($996–3895); upper middle income ($3896–12,055); and high income ($12,056 or more). Adjusted regression coefficients (B), confidence intervals (CIs), *p* values and partial eta squared (η^2^ as measure of effect size) were computed. Residual plots were assessed for the randomness of residuals’ distribution to ensure that model assumptions apply. Variance inflation factors were calculated to assess collinearity. Significance level was set at 5%. Statistical analyses were performed with SPSS version 22.0 (IBM Corp., Armonk, N.Y., USA).

## Results

ECC data were available for 39 countries for 0–2-year-olds and for 86 countries for 3–5-year-olds. Data on malnutrition and anemia were available for 128–185 countries, depending on malnutrition type. Combined ECC, anemia and malnutrition data were available for 26 countries in the age 0–2-years-old and 55 countries in the age 3–5-years-old (See Supplement 1 for list of countries). Thus, the results in this study are based on analysis of data from 26 and 55 countries in the 0–2-year-old group and the 3–5-year-old group, respectively.

The mean (SD) ECC prevalence was 23.8 (14.8)% in 0–2-year-old children and 57.3 (22.4)% in 3–5-year-old children. The overall mean (SD) prevalence of wasting was 6.3 (4.8)%, of overweight was 7.2 (4.9)%, of stunting was 24.3 (13.5)%, and of anemia was 37.8 (18.1)%.

The 26 countries that had complete data on ECC, anemia and malnutrition for 0–2-year-olds were two (7.7%) low-income countries, 10 (38.5%) lower middle-income countries, nine (34.6%) upper middle-income countries, and five (19.2%) high-income countries. The 55 countries that had complete data on ECC, anemia and malnutrition for 3–5-year-olds were six (10.9%) low-income countries, 19 (34.5%) lower middle-income countries, 22 (40%) upper middle-income countries, and eight (14.5%) high-income countries.

**Fig. 1:**
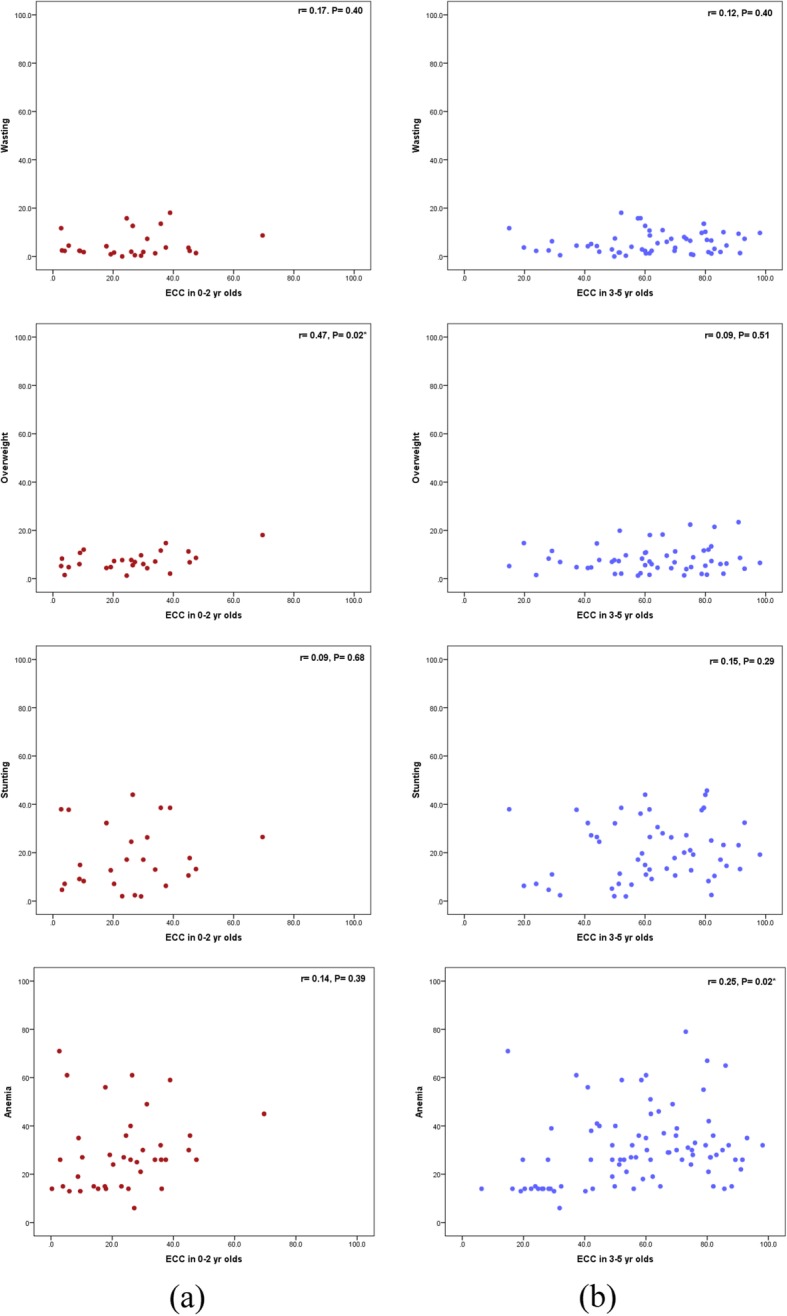
Scatter plots for the correlation between prevalence of malnutrition indicators, anemia and ECC in (**a**) 0–2 year-old children and (**b**) 3–5 year-old children

Figure 1(a) illustrates that ECC prevalence for 0–2-year-olds was positively, weakly and non-significantly correlated with the prevalence of wasting (r = 0.17, *P* = 0.40), stunting (r = 0.09, *P* = 0.68), and anemia (r = 0.14, *P* = 0.39). The prevalence of overweight was positively, moderately and significantly correlated with the prevalence of ECC (r = 0.47, *P* = 0.02).

Figure 1(b) reveals that among 3–5-year-olds, ECC prevalence was positively, weakly and non-significantly correlated with the prevalence of wasting (r = 0.12, P = 0.40), stunting (r = 0.15, *P* = 0.29), and overweight (r = 0.09, *P* = 0.51). However, the prevalence of anemia was positively, weakly and significantly correlated with the prevalence of ECC (r = 0.25, P = 0.02).

Results of the multivariate general linear analysis controlled for economic level are reported in Table 1. For children 0–2-years of age, the greatest effect size and only significant association was between the prevalence of ECC and the prevalence of overweight (η2 = 0.21); where countries with 1 % higher ECC prevalence had a 0.12% higher prevalence of overweight (B = 0.12, *P* = 0.03). For children 3–5 years of age, the greatest effect size and only significant association was between the prevalence of ECC and the prevalence of anemia (η2 = 0.08), where countries with 1 % higher prevalence of ECC had a 0.14% lower prevalence of anemia (B = − 0.14, *P* = 0.048). No collinearity was observed (variance inflation factors < 5).

**Table 1:** Association between prevalence of ECC in 0 to 2-year-olds and 3 to 5-year-olds, anemia and malnutrition

Malnutrition	ECC prevalence in 0–2 year olds			ECC prevalence in 3–5 year olds		
	B (95% CI)	P	η2	B (95% CI)	P	η2
Wasting	−0.02 (− 0.14, 0.11)	0.76	0.004	0.001 (−0.06, 0.06)	0.98	< 0.0001
Overweight	0.12 (0.02, 0.23)	0.03*	0.21	0.02 (−0.06, 0.09)	0.65	0.004
Stunting	−0.05 (− 0.31, 0.21)	0.68	0.008	−0.01 (− 0.14, 0.11)	0.84	0.001
Anemia	−0.15 (− 0.48, 0.19)	0.38	0.04	−0.14 (− 0.29, − 0.001)	0.048*	0.08

Multivariate general linear models were adjusted for income levels. For 0–2-year- olds: *p* value for income association with dependent variables < 0.05 except with overweight where *P* = 0.76. For 3–5-year-olds, p value for income association with dependent variables < 0.05 for all

η2: partial eta squared

*: statistically significant at *P* < 0.05

## Discussion

To our knowledge, this study provides the first evidence on the relationship between country-level prevalence of ECC, malnutrition, and anemia. However, not all types of malnutrition were associated with ECC. We found a positive and significant relationship between country-level prevalence of overweight and ECC in children 0–2-years of age, with higher prevalence of overweight associated with higher ECC prevalence. There was also an inverse relationship between country-level prevalence of anemia and ECC in 3–5-years olds with lower prevalence of anemia in countries with higher prevalence of ECC.

Overweight was associated with ECC only in children ≤2-years of age. The relationship between ECC, and childhood growth and development is not clear [5, 31]. The studies on the relationship between ECC and nutritional status provide conflicting results – some found no association [32–34], others found a positive association and some had inconclusive results [31, 35]. Recent Canadian studies reported that preschool children with severe ECC who were undergoing dental rehabilitation were more likely to have higher BMI z-scores than were caries-free controls [11, 12, 21, 23]. The inconsistent findings of the previous studies may be due to differences in the methods used for nutritional assessments, age range cut-offs, and confounders of dental caries, including differences in definition and severity of ECC [33].

The higher prevalence of overweight in countries with higher estimates of ECC in 0–2-year-olds may reflect the findings by El Tantawi et al. [26] who reported a higher prevalence of ECC in countries with greater economic growth. ECC and overweight/obesity share common risk factors – high frequency and quantity of free sugar consumption [36], food insecurity [37, 38], low socioeconomic status [39–41], residence in urban slums [42, 43] and rural areas [44, 45]. Growing economies are most likely to be undergoing nutrition transitions from traditional diets to low quality, processed, high-sugar, high-fat, carbohydrate-dense food and beverages that are poor in micronutrients [46, 47], which predisposes to overweight and high ECC prevalence. Our results might suggest that having ECC and being overweight have shared risk factors that are related to the macro-economic status of the country. A common risk factor approach [20, 48] may be used to address both ECC and overweight problems; with global action to control these health problems given priority to countries with greater economic growth.

The few studies that have assessed the relationship between ECC and nutritional status often included age ranges larger than that of ECC. Four studies conducted amongst preschool children, found no association between ECC and overweight in children 3 years of age [49], 2–5-year-olds [50], and 2–6-year-olds [51, 52]. These findings highlight the need for appropriate age groupings when studying ECC, as the relationship between ECC and nutritional status seems to be modulated by age. However, Davidson et al. [21] found that severe ECC was associated with obesity in 2–5-year-olds, thereby highlighting two additional dimensions to determining the association between ECC and overweight – the severity of ECC and the severity of overweight. Interestingly, those authors found an association between the two extremes of the phenomena studied. This finding also implies that enrolling children who have milder forms of caries and nutritional status may underestimate potential relationships [21]. Therefore, we suggest that future studies on ECC and nutritional status not only ensure that ECC is analyzed by age groups 0–2-year-olds and 3–5-year-olds, but also ensure that ECC and malnutrition data include the extremes of the variables, with emphasis on severe levels of ECC, such as those data using the World Health Organization Significant Caries Index. We exercise caution that the correlation we observed may be an artifact as correlational analysis at the macro-level is usually larger than it is for individuals [53].

Anemia, which is a complication of malnutrition and factors not malnutrition related [24, 36], was inversely associated with ECC in older preschool children. Anemia may not be a direct result of ECC, but it may be related to increased milk consumption in early childhood [23]. Evidence suggests that in developed countries where milk intake is high, the risk of anemia also is high [54, 55]. Anemia from high milk intakes results from early weaning of the child, and introduction of foods with low iron bioavailability. Milk also impairs non-heme and heme iron absorption [56]. Recent Canadian studies, a developed country that do not have malnutrition as a major health crisis [57] and have lower prevalence of ECC [58], reported that preschool children with severe ECC undergoing dental rehabilitation were more likely to have iron deficiency anemia than were caries-free controls [11, 12, 21, 23]. Future studies should explore this finding.

The present findings may have implications at the micro-level. Malnutrition is a complex disorder not solely caused by lack of food [59]. Feeding practices and other risk factors leading to malnutrition and/or anemia may be associated with greater risk for ECC. Health education programs to improve parental dietary choices and provision of sponsored healthful meals in kindergartens and similar gatherings may contribute to reducing the risk of these diseases but may not eliminate it. In addition, health providers who manage malnutrition, anemia and ECC could provide better and more comprehensive care to children by screening for either of the diseases and referring children to for specialized care. Structuring primary health care services to provide integrated dental and pediatric care may help address the dual burden of ECC and malnutrition. Dentists who care for patients with severe ECC should be aware that the children may have undiagnosed nutritional disease that warrants investigation.

One of the strengths of this study is that collated data on malnutrition included high-quality data from the Demographic Health Survey [60]. However, there is the risk of over-representation of children who have living mothers since the anthropometric variables used for assessment of nutritional status are only available for those who are alive: the sample may therefore have under-represented malnutrition in infants and preschool children [61, 62]. We controlled for gross national income per capita, but we could not control for all possible confounders, as these remain largely unknown due to lack of data. One of the confounders is sex, which plays a role in nutrition [63], but its role as a risk factor for ECC is unknown. We also were not able to adjust for possible confounders like sugar intake and oral hygiene status as country-level data for these variables were not available. Controlling for these factors may attenuate the relationships we established in this study. Our use of the z-scores adjusted for age and sex to determine nutritional status allowed for more meaningful reporting of means [21]. We did not use the body mass index to assess nutritional status because it is meant to be used in children ≥2 years of age whereas we focused on 2-year-olds and younger, in addition to children > 2 years of age.

A limitation of this study was use of the World Health Organization’s criteria for assessing caries by many of the epidemiological surveys [64]. This assessment tool does not include non-cavitated lesions: only 15% of ECC surveys reported non-cavitated and/or cavitated as the caries detection level [36]. The ECC prevalence for many countries may therefore be under-reported. Our study analysis was also limited by only a minor portion of the global ECC prevalence estimates being based on national surveys, which made generalizability of the study findings challenging. ECC is under-studied in many parts of the world and true population estimates are often unknown. Also, although we split the data on ECC into two age groups - 0-2 and 3–5-year-olds – estimates for wasting, stunting and overweight could not be split because the data were not available. Further, our study is cross-sectional, so the direction of the observed relationships cannot be ascertained i.e., whether ECC increases the risk of 0–2-year-old children being overweight, or being overweight increases the risk for ECC. A carefully designed longitudinal study may answer the questions this study have raised but were unanswered.

## Conclusion

Country-level prevalence of ECC was associated with malnutrition in 0–2-year-olds and with anemia in 3–5-year-olds. We also observed age-related disparities in the relationship between ECC, malnutrition, and anemia. The disparities reiterate the need for future analysis of ECC-related data for the two age groups as earlier highlighted by El Tantawi et al. [26]. Further, prospective studies are needed to determine how various social, economic and cultural factors that characterize these age groups impact the co-morbid existence of the three diseases. Though prior research findings suggests that the pathway for the direct relationship between ECC and overweight may be diet related, the pathway for the inverse relationship between ECC and anemia is less clear and needs further investigations The small magnitude of the associations suggest that other factors may modulate the association between ECC, anemia and overweight. Longitudinal studies assessing the relationship between separate age groups, ECC severity and severity of malnutrition is necessary to understand the relationship between ECC, malnutrition, and anemia. Understanding these relationships might aid in the designing of cost-effective and efficient global preventive interventions using the common risk factor approach for infants and preschool children.

## Supplementary information

**Additional file 1.** Countries with complete data on early childhood caries, anemia and malnutrition.

## Data Availability

Study related data and materials are enclosed as supplementary file. The complete details on the ECC prevalence by age group and country is available as a supplementary file of the publication by: El Tantawi M, Folayan MO, Mehaina M, Vukovic A, Castillo JL, Gaffar BO, et al. Prevalence and Data Availability of Early Childhood Caries in 193 United Nations Countries, 2007–2017. American journal of public health. 2018; 108(8):1066–1072. The nutrition related data is accessible at: https://data.unicef.org/topic/nutrition/malnutrition/. Published 2018. Updated May 2018. The anemia related data is accessible at: WHO. The global prevalence of anemia in 2011. https://www.who.int/nutrition/publications/micronutrients/global_prevalence_anaemia_2011/en/. Published 2015.

## Abbreviations

CI: Confidence Interval
ECC: Early Childhood Caries
UNICEF: United Nations Children’s Emergency Funds
SD: Standard Deviation
WHO: World Health Organization
