# Supplementary material for: Associations between early childhood caries, malnutrition and anemia: a global perspective
Source: BMC Nutr. 2020 May 4;6:16. doi: 10.1186/s40795-020-00340-z (PMC7197144; doi:10.1186/s40795-020-00340-z)
Supplement: Supplementary file 1 — Additional file 1. Countries with complete data on early childhood caries, anemia and malnutrition. [file 40795_2020_340_MOESM1_ESM.docx]

**ASSOCIATIONS BETWEEN EARLY CHILDHOOD CARIES AND MALNUTRITION AND ANEMIA: A GLOBAL PERSPECTIVE**

**Supplementary file 1**

**Countries with complete data on early childhood caries, anemia and malnutrition in 0-2-year-old children (n= 26)**

1. Australia
2. Brazil
3. Chile
4. China
5. Colombia
6. Ecuador
7. Egypt
8. El Salvador
9. India
10. Indonesia
11. Japan
12. Kazakhstan
13. Kuwait
14. Kyrgyzstan
15. Mexico
16. Mongolia
17. Morocco
18. Namibia
19. Nigeria
20. Pakistan
21. Paraguay
22. Serbia
23. Sri Lanka
24. Tanzania
25. Uganda
26. United States

**Countries with complete data on early childhood caries, anemia and malnutrition in 0-2-year-old children (n= 55)**

1. Albania
2. Australia
3. Bosnia & Herzegovina
4. Brazil
5. Brunei
6. Cambodia
7. Chile
8. China
9. Colombia
10. Congo, DRC
11. Ecuador
12. Egypt
13. El Salvador
14. Gambia
15. Georgia
16. India
17. Indonesia
18. Iran
19. Iraq
20. Jamaica
21. Japan
22. Kazakhstan
23. Kenya
24. Kuwait
25. Kyrgyzstan
26. Laos
27. Libya
28. Malaysia
29. Mexico
30. Mongolia
31. Morocco
32. Myanmar
33. Namibia
34. Nepal
35. Nigeria
36. North Sudan
37. Pakistan
38. Paraguay
39. Peru
40. Philippines
41. Senegal
42. Serbia
43. South Africa
44. South Korea
45. Sri Lanka
46. Syria
47. Tanzania
48. Thailand
49. Trinidad & Tobago
50. Turkey
51. Uganda
52. United States
53. Vanuatu
54. Venezuela
55. Vietnam
